# 17α-Ethynylestradiol and Levonorgestrel Exposure of Rainbow Trout RTL-W1 Cells at 18 °C and 21 °C Mainly Reveals Thermal Tolerance, Absence of Estrogenic Effects, and Progestin-Induced Upregulation of Detoxification Genes

**DOI:** 10.3390/genes15091189

**Published:** 2024-09-10

**Authors:** Margarida Vilaça, Célia Lopes, Rosária Seabra, Eduardo Rocha

**Affiliations:** 1 Laboratory of Histology and Embryology, Department of Microscopy, ICBAS–School of Medicine and Biomedical Sciences, University of Porto (U.Porto), Rua Jorge Viterbo Ferreira 228, 4050-313 Porto, Portugal; up201804847@edu.fc.up.pt (M.V.); rcseabra@icbas.up.pt (R.S.); erocha@icbas.up.pt (E.R.); 2 Team of Animal Morphology and Toxicology, CIIMAR/CIMAR–Interdisciplinary Centre of Marine and Environmental Research, University of Porto (U.Porto), Terminal de Cruzeiros do Porto de Leixões, Av. General Norton de Matos s/n, 4450-208 Matosinhos, Portugal

**Keywords:** endocrine disruption, estrogens, fish cell lines, mixtures, progestins, climate change

## Abstract

Fish are exposed to increased water temperatures and aquatic pollutants, including endocrine-disrupting compounds (EDCs). Although each stressor can disturb fish liver metabolism independently, combined effects may exist. To unveil the molecular mechanisms behind the effects of EDCs and temperature, fish liver cell lines are potential models needing better characterisation. Accordingly, we exposed the rainbow trout RTL-W1 cells (72 h), at 18 °C and 21 °C, to ethynylestradiol (EE2), levonorgestrel (LNG), and a mixture of both hormones (MIX) at 10 µM. The gene expression of a selection of targets related to detoxification (*CYP1A*, *CYP3A27*, *GST*, *UGT*, *CAT*, and *MRP2*), estrogen exposure (*ERα*, *VtgA*), lipid metabolism (*FAS*, *FABP1*, *FATP1*), and temperature stress (*HSP70b*) was analysed by RT-qPCR. *GST* expression was higher after LNG exposure at 21 °C than at 18 °C. LNG further enhanced the expression of *CAT*, while both LNG and MIX increased the expressions of *CYP3A27* and *MRP2*. In contrast, *FAS* expression only increased in MIX, compared to the control. *ERα*, *VtgA*, *UGT*, *CYP1A*, *HSP70b*, *FABP1*, and *FATP1* expressions were not influenced by the temperature or the tested EDCs. The RTL-W1 model was unresponsive to EE2 alone, sensitive to LNG (in detoxification pathway genes), and mainly insensitive to the temperature range but had the potential to unveil specific interactions.

## 1. Introduction

Aquatic ecosystems have been the sink for many contaminants that reach them through direct discharges or sewages [1,2]. Among these contaminants are the endocrine-disrupting chemicals (EDCs), which interfere with the homeostasis and normal functioning of the endocrine system [3]. Natural and synthetic steroid hormones, like estrogens, progestogens, and androgens, belong to EDCs [2] and are commonly found in aquatic ecosystems [4]. Due to their massive pharmaceutical use, the synthetic estrogen 17α-ethynylestradiol (EE2) and progestins like levonorgestrel (LNG), norethindrone (NET), or megestrol acetate (MTA) are ubiquitous pollutants in aquatic ecosystems [5,6,7,8]. EE2 was even included in the European Union Watch list under the Water Framework Directive [9]. EDCs can disrupt essential physiological processes controlled by steroid hormones, including development [6,10], growth [11], and reproduction [11,12]. One example of such disruptions is the abnormal production of vitellogenin (Vtg), the precursor of oocyte yolk proteins. Vtg synthesis is naturally induced in oviparous female fish liver by estradiol (E2), a process genomically mediated through estrogen receptors (ERs) [13,14], despite the existence of lesser-known non-genomic effects [15,16]. Significant Vtg induction, particularly in juveniles and males (which have minimal E2 levels), is considered a robust biomarker of exposure to environmental estrogens [14]. Natural and synthetic progestogens, e.g., LNG, NET, and progesterone (P4), can also interfere with Vtg synthesis, usually decreasing its mRNA and protein levels [17,18,19].

Even though the literature is most extensive regarding EE2’s effects related to reproduction, some studies have shown that this compound can also impact lipid metabolism by decreasing triglyceride storage and xenobiotic metabolism in fish liver [6,20,21]. The same has been evidenced for detoxification genes. For example, cytochrome P450 (CYP) 1A1 mRNA levels and enzymatic activity were decreased in Atlantic salmon (*Salmo salar*) exposed to 5 and 50 ng/L of EE2 [22]; the expression of other biotransformation-related enzymes was not altered. Anyway, the disturbance of detoxification enzymes’ expression and activity can lead to an increased production of reactive oxygen species (ROS) [23], culminating in high levels of oxidative stress [24]. As for progestogens, effects on targets other than the reproductive system have been very scarcely described. Nevertheless, it was reported in a transcriptome study that LNG (0.3 µg/L) can deregulate the lipid metabolism, increasing the expression of lipid biosynthesis and transport genes in female and male Eastern mosquitofish (*Gambusia holbrooki*) liver, respectively [25].

In addition to the threat of EDCs pollution, marine and freshwater ecosystems face the challenges of multiple stressors. Extreme weather events such as heavy rainfalls, floods, heat waves, and droughts are becoming more frequent, and global warming is increasing water temperature and decreasing water quality, with changes in abiotic factors such as salinity and dissolved oxygen [26,27]. Most fish are ectothermic, meaning they cannot control their body temperature through metabolic activity [28]. As a result, an increase in water temperature has a significant effect on their physiology, as this abiotic factor is essential to many processes, including liver metabolism [29,30] and reproduction [31,32]. Also, increased temperatures have been shown to increase oxidative stress and disrupt metabolic pathways related to energy production in fish liver [30,33,34]. 

The independent effects of increased temperature and exposure to EDCs have been fairly well characterised in fish. However, in nature, aquatic organisms are affected by complex mixtures of contaminants and continuously higher temperatures. This is relevant since the effects of mixtures of estrogenic compounds [35,36] and even of compounds with different mechanisms of action [37] can be more detrimental than those of the same compounds in single exposures. In line with this, additive (estrogenic) effects of EE2 and LNG on the *CYP19A1B* gene expression in the brain were reported in zebrafish (*Danio rerio*), in vivo and in vitro [38]. In vitro, LNG alone did not activate the expression of the luciferase gene under the control of the zebrafish *CYP19A1B* promoter, but activation was achieved when LNG was mixed with EE2 and in a concentration-dependent manner [38]. Additive effects were also observed in the reproductive system of zebrafish after exposure to EE2 (10 ng/L) and the progestin MTA (33, 100, and 333 ng/L) [39]. EE2 + MTA significantly decreased steroid hormone levels (compared to MTA alone), downregulated the expression of genes involved in steroid production, maturation, and ovulation (compared to the solvent control group), and reduced egg production (compared to EE2 alone) [39].

Combined effects of both temperature and contaminants can also arise. High temperatures can alter the chemical degradation and toxicity of aquatic pollutants, consequently affecting how organisms respond to these contaminants [40,41]. It is reported that a high temperature exacerbates the effects of contaminants [42,43], including EDCs [19]. For example, the hepatocytes from female zebrafish exposed to LNG (10 ng/L and 1000 ng/L) at 30 °C contained more glycogen than the ones exposed at 27 °C [19]. Further, the immunohistochemical analysis revealed a more pronounced reduction in Vtg in fish exposed to LNG (10 ng/L) at 30 °C compared with that of the 27 °C groups. Also, in the inland silverside (*Menidia beryllina*), an euryhaline model fish, EE2 and bifenthrin had more pronounced effects in sex determination and early development at the highest tested temperature (28 °C versus 22 °C) [44].

Despite the described evidence of mixed effects of temperature and toxicants, including EDCs, this subject remains relatively unexplored. In vivo and in vitro strategies may be employed to deepen the knowledge of this matter. For mechanistic studies, liver in vitro models can be even more worthy since the confounding factors present in in vivo models are eliminated [45]. The RTL-W1 is a normal liver-derived cell line obtained from rainbow trout (*Oncorhynchus mykiss*) [46]. This species, like other salmonids, is widely distributed in freshwater ecosystems, especially those of the Northern Hemisphere. It represents an important species for aquaculture worldwide and is one of the most used in ecotoxicological and biomonitoring studies [47]. The cell line preserves some hepatocytic features [46,48], expresses biotransformation enzymes [46,49], and was considered suitable for studying the “toxicity of complex or unknown mixture of chemicals” [50]. Still, there are virtually no articles on RTL-W1 cells’ ability to respond to estrogens and no data about progestogens’ effects. According to Fent [51], Ackermann et al. (unpublished) found that RTL-W1 had mRNA of *ER* and *Vtg*, but they were not upregulated by E2. In an exploratory study, RTL-W1 cells were exposed to E2 and EE2, which did not upregulate *ERα1* despite causing ultrastructural changes; the findings were not extended or published [52]. Finally, the weak xenoestrogen bisphenol-A (BPA) increased RTL-W1 cells’ triacylglycerols [53].

In this study, our goal was to first assess if this cell line, cultured in monolayer (2D), can be used as a model to study the isolated and combined effects of temperature and EDCs on the liver of fish, especially from the *Salmonidae* family. We thus exposed the cell line to an estrogen (EE2) and a progestin (LNG) at 18 °C and 21 °C. The 18 °C condition is normothermia for rainbow trout hepatocytes [54], and the 21 °C condition is realistically higher, e.g., within the range from +1.0 to +3.9 °C of increase predicted for rivers in the Iberian Peninsula throughout the 21st century [55] and for freshwater ecosystems across Europe and North America, with expected increases between 1 and 3 °C [26]. After exposures, we assessed the effects of both stressors on the cell viability and gene expression of targets related to detoxification (*CYP1A*, *CYP3A27*, *glutathione S-transferase omega 1* (*GST*), *uridine diphosphate* (*UDP*)-*glucuronosyltransferase* (*UGT*), *catalase* (*CAT*), and *multidrug resistance-associated protein 2* (*MRP2*)), estrogen-exposure (*ERα*, *VtgA*), lipid metabolism (*fatty acid synthase* (*FAS*), *fatty acid binding protein 1* (*FABP1*), *fatty acid transport protein* (*FATP1*)), and temperature-related stress (*heat shock protein 70b* (*HSP70b*)). In addition to testing the RTL-W1 model in the studied context, the new data help to further characterise the cell line regarding the expression of a variety of genes and better understand the consequences of the mixed effects of temperature and EDCs in fish liver, knowledge of utmost importance in the current global warming scenario.

## 2. Materials and Methods

### 2.1. RTL-W1 Cell Culture

The RTL-W1 cells were routinely cultured on gas-permeable T75 culture vessels (5520200, Orange Scientific, Braine-l’Alleud, Belgium) with 15 mL of phenol red-free Leibovitz’s (L-15) culture medium (21083-027, Gibco^TM^, Thermo Fisher Scientific, Grand Island, NY, USA), supplemented with 5% fetal bovine serum (FBS) (*v*/*v*) (F9665, Sigma-Aldrich, St. Louis, MO, USA) and 1% penicillin/streptomycin (10,000 U/10,000 µg/mL) (A2213, Merck KGaA, Darmstadt, Germany), in a refrigerated incubator (Heraeus, BK 6160, Thermo Scientific, Langenselbold, Germany) at 18 °C, without CO_2_ atmosphere. The medium was replaced thrice a week until the flasks became confluent. For the subculture, the cells were detached using 0.05/0.02% trypsin/ethylenediaminetetraacetic acid (EDTA) (59418C, Sigma-Aldrich, St. Louis, MO, USA) and passed to new T75 culture vessels. All the cell culture procedures and the later protocol steps were strictly standardised to minimise the inherent inter-assay variability occurring even for “highly standardised cell culture experiments” [56].

### 2.2. Exposure to EDCs

To assess the RTL-W1 cell line potential for studying the combined effects of temperature and EDCs, the cells were exposed to EE2 (CAS 57-63-6, E4876, Sigma-Aldrich, St. Louis, MO, USA) and LNG (CAS 797-63-7, PHR1850, Sigma-Aldrich, St. Louis, MO, USA) at 18 °C (incubator Heraeus BK 6160, Thermo Scientific, Langenselbold, Germany) and 21 °C (incubator INCU-LINE^®^ 150R PREMIUM, VWR International, Leuven, Belgium). For each temperature, five independent experiments (different days) were conducted using passages between 107 and 121. Each experiment comprised three 24-well plates (4430300, Orange Scientific, Braine-l’Alleud, Belgium), in which 80,000 cells were seeded per well in 500 µL of L-15 medium supplemented with 5% charcoal-stripped FBS (*v*/*v*) (F6765, Sigma-Aldrich, St. Louis, MO, USA) and 1% penicillin/streptomycin (10,000 U/10,000 µg/mL) (A2213, Merck KGaA, Darmstadt, Germany).

Stock solutions for exposures were prepared in dimethylsulfoxide (DMSO) (0231-500ML, VWR, Solon, OH, USA). Then, each plate was designed to encompass five different conditions: control (C)—supplemented L-15 medium; solvent control (SC)—0.1% DMSO in supplemented L-15 medium; EE2—10 µM of EE2; LNG—10 µM of LNG; and a mixture (MIX) consisting of 10 µM of EE2 + 10 µM of LNG. Exposure solutions were prepared from the stocks in supplemented L-15 medium to a final 0.1% DMSO concentration (except C). The steroid concentrations were selected based on the reported ranges for EE2 and other estrogenic EDCs, as they were able to change the expression of some of the selected targets in the RTL-W1 cell line [53], rainbow trout [57], and brown trout (*Salmo trutta*) [58] primary hepatocytes. The different experimental conditions were randomly allocated to the plate wells, so each well had the same probability of being assigned to a condition. The cells were allowed to adhere for 24 h and then exposed to different conditions for 72 h, replacing the solutions daily.

At the end of the exposures, the cells were trypsinised and counted, and their viability was assessed in three wells per condition/plate using the trypan blue exclusion assay [59]. Briefly, 10 µL of the cell suspension was mixed with 10 µL of 0.4% trypan blue in PBS. The total viable and death cells (that internalise the dye due to membrane damage) were counted in a Neubauer chamber (0640031, Marienfeld, Lauda-Königshofen, Germany) to calculate cell density and viability. A minimum of four wells of the same condition per plate were pooled for gene expression analyses (summing three pellets per condition, one from each plate). The cells were pelleted by centrifugation (200× *g*, 5 min at 15 °C; 521-1895 Mega Star, VWR International, Leuven, Belgium) and then frozen in liquid nitrogen (after removing the supernatant) and kept at −80 °C until RNA extraction was performed.

### 2.3. RNA Extraction and cDNA Synthesis

The total RNA was extracted and purified from the cell pellets using the illustra™ RNAspin Mini Isolation Kit (Cat. No. 25-0500-72, GE Healthcare, Amersham, UK). The isolated RNA integrity was confirmed by electrophoresis in a 1% agarose gel stained with GelRed (Biotium, Fremont, CA, USA) and quantified using the Multiskan Go spectrophotometer (Thermo Fisher Scientific Oy, Vantaa, Finland) using the μDrop™ Plate (Thermo Fisher Scientific, Singapore); 260/280 and 260/230 ratios were determined to evaluate the purity of the nucleic acid samples. As mRNA quality was consistent among plates, two replicates per condition, per independent experiment, were randomly chosen to synthesise cDNA. In total, 500 ng of the total RNA of those samples was transcribed into cDNA using the iScript™ Reverse Transcription Supermix for RT-qPCR (1708841, Bio-Rad, Hercules, CA, USA).

### 2.4. Gene Expression Analysis

The expression of the selected genes was analysed by reverse transcription–quantitative polymerase chain reaction (RT-qPCR) using the CFX Connect™ (Bio-Rad, Hercules, CA, USA) thermal cycler, following the detailed amplification protocols listed in Table 1. For all primer pairs, calibration curves were performed before the RT-qPCR experiments to calculate the efficiencies of the reactions for each primer pair.

Each RT-qPCR plate contained duplicates of each sample, no template controls (NTC) where cDNA was substituted with nuclease-free water, and a calibrator (a mixture of cDNA from three randomly selected samples). The reaction mixtures (total volume: 20 μL) consisted of 10 μL of iQ™ SYBR^®^Green Supermix (1708886, Bio-Rad, Hercules, CA, USA), 200 nM of specific primers for each gene (Table 1), and 5 μL of diluted (1:10 in nuclease-free water) cDNA. At the end of the amplification cycles, a melting curve was plotted—from 55 °C to 95 °C, with 0.5 °C increments, for 30 s per step—to verify the specificity of the reaction product.

The *ef1α* and *β-actin* were steadily expressed at both temperatures and between exposure conditions and then used as reference genes for the relative quantification of mRNA levels by the Pfaffl method [69].

### 2.5. Statistical Analysis

Descriptive and inferential analyses were performed using Jamovi (Version 2.3) [70] software, and graphs were plotted using GraphPad Prism 9.0.0 [71]. The relative mRNA levels obtained from the two plates of each independent experiment were averaged so each assay could be considered as the experimental unit (*n* = five independent experiments per temperature). The data were tested for parametric assumptions of normality and homogeneity of variances, performing the Shapiro–Wilk W and Levene’s tests, respectively. Then, they were analysed by two-way ANOVA, followed by the post hoc Tukey’s test. The data were transformed into logarithms, inverses, square roots, and ranks when necessary to accomplish this parametric test and achieve normality and homogeneity of variances. There were no significant differences between the data from the C and SC conditions, so they were treated as only one group (C condition) in the statistical analysis [72]. Differences were considered significant whenever *p* < 0.05. A principal component analysis (PCA) was additionally conducted to determine the components that relevantly account for most of the gene data’s variability while examining the extension on the basis of which the different experimental groups could be distinguished in the PCA Scatter Plot. The number of relevant principal components was investigated using the Kaiser criterion (i.e., by selecting those with eigenvalues larger than 1) but in conjunction with the broken stick rule. The PCA adopted the correlation matrix mode and was implemented with the software PAST (4.17) [73].

## 3. Results

### 3.1. Cell Density and Viability

**Figure 1:**
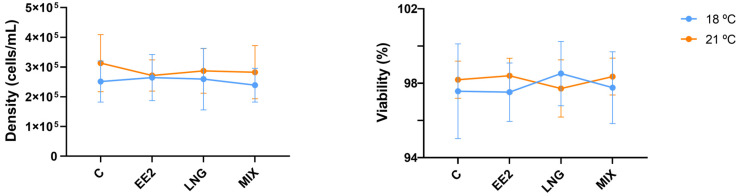
Cell density and viability after 72 h of exposure to 17α-ethynylestradiol (EE2) and levonorgestrel (LNG) at 18 °C and 21 °C. C—control; EE2—10 µM of EE2, LNG—10 µM of LNG, and MIX—10 µM of EE2 + 10 µM of LNG. Data are presented as the mean ± standard deviation. *n* = 5 independent experiments per temperature.

In all the exposure groups, the cell density was consistently ≥2.39 × 10^5^ cells/mL, while the cell viability remained ≥97.5%, regardless of the temperature. There were no significant differences among experimental groups or temperatures (Figure 1).

### 3.2. Gene Expression 

**Figure 2:**
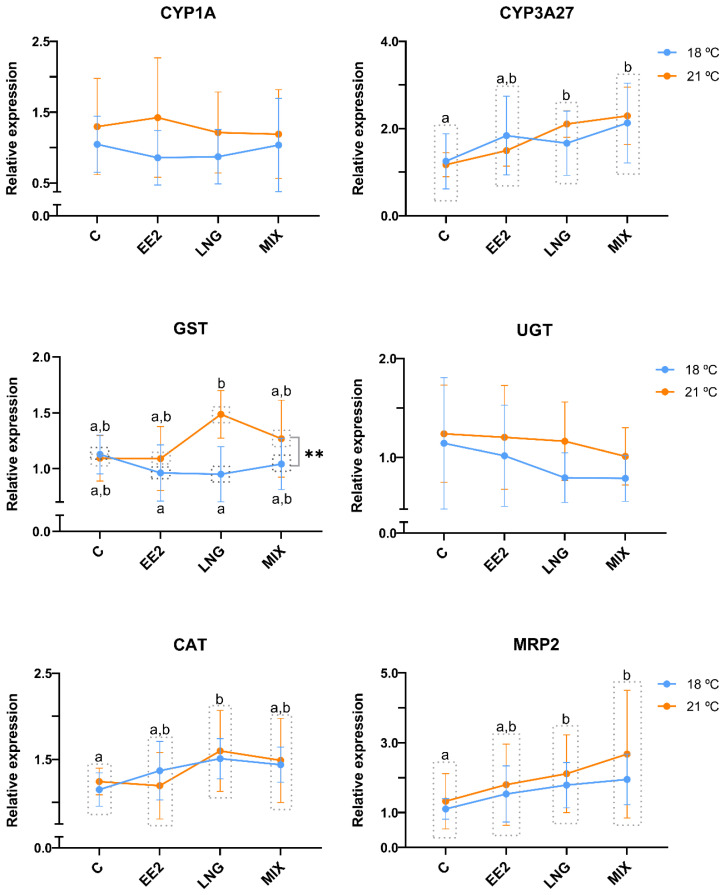
Relative gene expression of targets related to detoxification (*CYP1A*, *CYP3A27*, *CAT*, *GST*, *UGT*, *MRP2*) in RTL-W1 cells after 72 h of exposure to 17α-ethynylestradiol (EE2) and levonorgestrel (LNG) at 18 °C and 21 °C. C—control; EE2—10 µM of EE2, LNG—10 µM of LNG, and MIX—10 µM of EE2 + 10 µM of LNG. Data concerning *CYP1A* (*Cytochrome P450 1A*), *CYP3A27* (*Cytochrome P450 3A27*), *CAT* (*Catalase*), *GST* (*Glutathione S-transferase omega 1*), *UGT* (*Uridine diphosphate* (*UDP*)*-glucuronosyltransferase*), and *MRP2* (*Multidrug Resistance-Associated Protein 2*) relative expression levels are presented as the mean ± standard deviation. Different letters illustrate significant differences between conditions, according to two-way ANOVA followed by Tukey’s test, and the symbol ** denotes significant differences (*p* < 0.01) between temperatures. *n* = 5 independent experiments per temperature.

**Figure 3:**
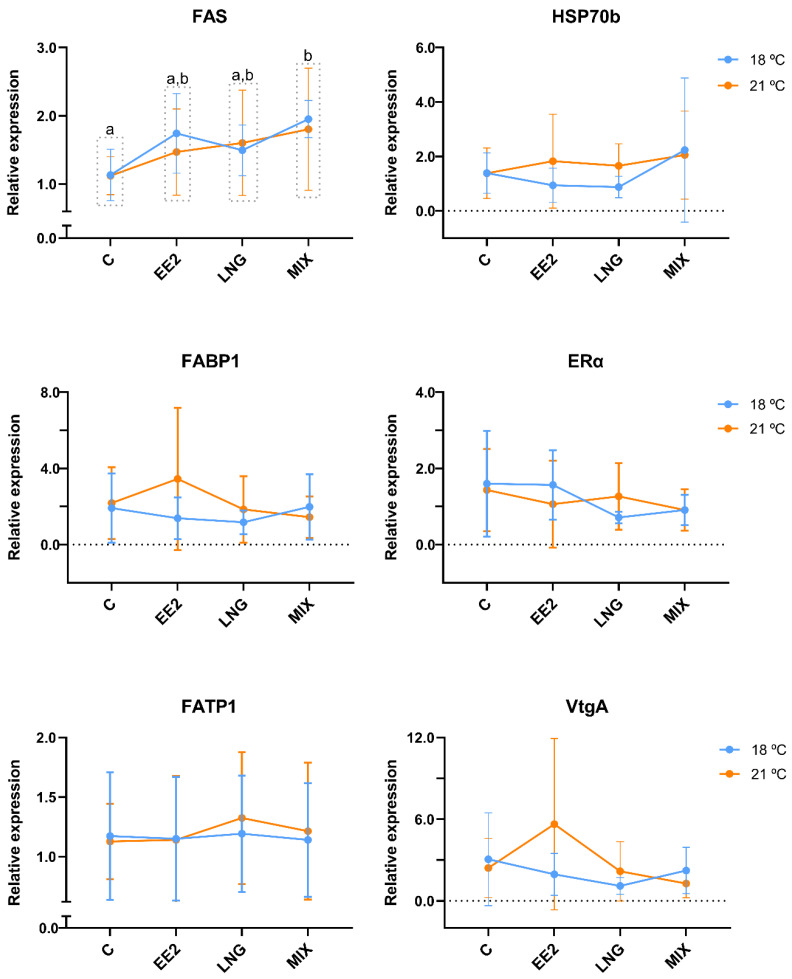
Relative gene expression of targets related to lipid metabolism (*FAS*, *FABP1*, *FATP1*), estrogenic effects (*ERα*, *VtgA*), and temperature-related stress (*HSP70b*) in RTL-W1 cells after 72 h of exposure to 17α-ethynylestradiol (EE2) and levonorgestrel (LNG) at 18 °C and 21 °C. C—control; EE2—10 µM of EE2, LNG—10 µM of LNG, and MIX—10 µM of EE2 + 10 µM of LNG. Data concerning *FAS* (*Fatty Acid Synthase*), *FABP1* (*Fatty Acid Binding Protein 1*), *FATP1* (*Fatty Acid Transport Protein 1*), *HSP70b* (*Heat Shock Protein 70 b*), *ERα* (*Estrogen Receptor α*), and *VtgA* (*Vitellogenin A*) relative expression levels are presented as the mean ± standard deviation. Different letters illustrate significant differences between conditions, according to two-way ANOVA followed by Tukey’s test. *n* = 5 independent experiments per temperature.

The relative gene expression of RTL-W1 cells exposed at 18 °C and 21 °C to EE2 and LNG is represented in Figure 2 and Figure 3. Regarding the detoxification-related genes (Figure 2), neither the exposure to the compounds nor the temperature influenced the relative expression of *CYP1A*. As for the other phase I detoxification enzyme, *CYP3A27*, the two-way ANOVA results showed a significant increase in its relative mRNA expression after exposure to LNG and MIX (LNG, *p* = 0.022; MIX, *p* = 0.001) compared to group C. Regarding detoxification phase II genes, *UGT*’s expression was not altered by temperature or the tested compounds, while *GST*’s expression differed among temperatures, being more expressed at 21 °C than at 18 °C (*p* = 0.004). Additionally, an interaction effect between EDCs and temperature effects was observed on this gene’s expression. Specifically, LNG at 18 °C significantly differed from LNG at 21 °C (*p* = 0.018). The expression of *MRP2* increased after LNG and MIX (LNG, *p* = 0.049; MIX, *p* = 0.012), while *CAT*’s expression was significantly increased by exposure to LNG (*p* = 0.017) compared with group C. The temperature did not significantly influence the mRNA levels of both *MRP2* and *CAT*.

Concerning the genes involved in lipid metabolism (*FAS*, *FABP1*, and *FATP1*), only *FAS* expression was altered, being significantly increased by exposure to MIX (*p* = 0.006) compared to C (Figure 3). Finally, the cell line expressed the genes *ERα* and *VtgA* (typically estrogen-responsive) and *HSP70b* (temperature-related stress), but their expression was not significantly different at 18 °C and 21 °C or influenced by the exposure to EE2, LNG, or MIX (Figure 3).

**Figure 4:**
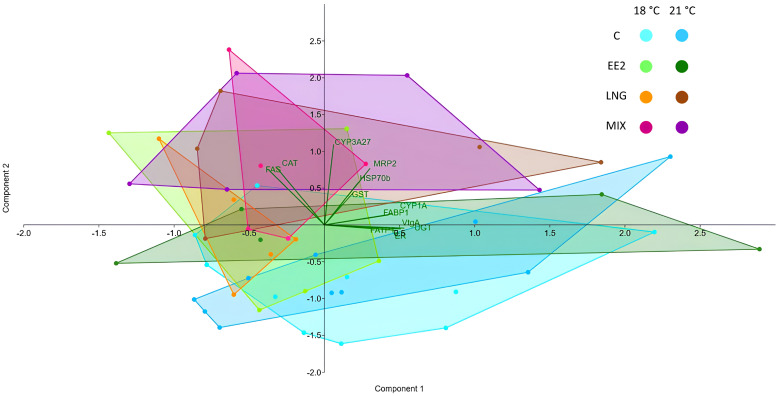
Principal component analysis scatter plot for all the experimental groups (each depicted with a convex hull, distinctly filled with unique colours). C—Control; 17α-ethynylestradiol (EE2)—10 µM; levonorgestrel (LNG)—10 µM, and MIX—10 µM of EE2 + 10 µM of LNG at 18 °C and 21 °C.

Regarding the PCA results, the scatter plot (Figure 4) aligned with the inferences from the ANOVA. From the plot, there is no substantial clustering of groups given the temperature effect, except for the exposure to LNG, where the 18 °C vs. 21 °C almost clustered entirely. The groups exposed to MIX and LNG tended to broadly overlap, consistent with the fact that most of the significant differences in ANOVA were found under those exposures. Similarly, the groups C and EE2 largely overlapped, with the former being quite distinguishable from the LNG and MIX groups and the EE2 overlapping with all. The PCA identified that the first three components explained 69.5% of the variance (PC1—38.7%, eigenvalue 4.6; PC2—18.5%, eigenvalue 2.2; PC3—12.3%, eigenvalue 1.5, but not relevant according to the broken stick rule). The higher PC1-positive loadings for the variability were due to *UGT*, *CYP1A*, *FABP1*, *ERα*, and *VtgA* (all not revealing significant differences in ANOVA). The higher PC2-positive loadings for the variability were due to *MRP2*, *CAT*, *GST*, *CYP3A27*, *FAS*, and *HSP70b* (only the latter did not show differences in ANOVA).

## 4. Discussion

Neither EE2 nor LNG influenced the density and viability of the cells, indicating that neither the compounds nor the tested temperatures were toxic or lethal to the RTL-W1 cells in our experimental set-up or significantly impacted cell survival and the potential ability to function in the culture. Regarding EE2, our results align with those of Madureira et al. [58], who observed that brown trout primary hepatocytes were also not significantly impacted by EE2 exposure regarding density and viability, even at higher concentrations (50 µM) and exposure times (72 and 96 h). To our knowledge, no studies have yet examined the in vitro effects of LNG on cell viability, particularly concerning fish primary hepatocytes or liver-derived cell lines. It cannot be excluded, however, that higher drug concentrations, longer exposure times, or exposure to a higher temperature could have impacted cell viability. In primary rainbow trout hepatocytes cultured at 10, 15, 20, and 25 °C, cell viability was shown not to differ between conditions during the first 6 days in the culture. However, a significant decrease in survival was observed in the 25 °C condition after 8, 10, and 14 days in the culture [74].

Here, in the tested conditions, the RTL-W1 cell line was non-responsive to EE2 (10 µM) alone. The RTL-W1 cell line expresses low mRNA levels of *ERα*; however, in 72 h single exposures, EE2 did not alter its relative mRNA expression or that of any of the studied genes, including Vtg, the most robust biomarker of estrogen exposure [14]. Backing up our result, the RTL-W1 cell line, when exposed (only in duplicate) for 48 h to 1 and 10 µM of E2 (in monolayer) and for 96 h to 10 µM of EE2 (in cell aggregates), expressed low but stable levels of *ERα1* mRNA [52]. Nevertheless, EE2 exposure induced ultrastructural changes, markedly increasing the number of cytoplasmic filaments. The appearance of cytoplasmic intermediate filaments was also induced after E2 exposure in Armenian hamster primary hepatocytes [75]. Earlier in the RTL-W1 cell line, it was stated that ER mRNA and Vtg (mRNA and protein) were not inducible by E2, but no methods or data were published [51]. The loss of differentiation and responses to particular stimuli is common when using cell lines in experiments. For example, in other cell lines, namely, the ZF-L (zebrafish liver) and RTH-149 (from rainbow trout hepatoma), Vtg production also did not exhibit a dose-dependent response after stimulation by E2 (1 to 10 µM) for 48 h, secreting less Vtg than brown trout primary hepatocytes [76].

Here, temperature also did not impact the levels of *VtgA* in RTL-W1 cells. Eventually, this result could be thought of as only related to the low expression levels of *ERs and Vtg* in the RTL-W1 cell line. However, our finding aligns with the behaviour of primary hepatocytes from rainbow trout held for 48 h and 96 h at 14 and 18 °C, in which temperature alone did not influence the estrogenic potential [77]. Still, it cannot be excluded that exposure to higher temperatures or different exposure lengths could increase Vtg levels, as verified in vivo, in Atlantic salmon juveniles [78] and immature female Japanese eels (*Anguilla japonica*) [79] kept at different temperatures.

Herein, there were also no alterations in *Vtg* mRNA levels after LNG exposure, differing from studies with female fathead minnow [80], using P4, and female zebrafish [19], using LNG, where the Vtg mRNA and protein levels fell. LNG (16 ng/L) also decreased *ERα* mRNA levels in fathead minnow larvae after 28 days [81], reinforcing an antagonistic effect of LNG on estrogen signalling. However, high LNG concentrations (3124 ng/L) exerted estrogenic effects in the roach (*Rutilus rutilus*), increasing *Vtg* and *ERα* mRNA levels in males and females [82] and raising the hypothesis of non-monotonic effects of LNG on estrogenic signalling.

Although there was a general absence of a response of the cell line to EE2, a different pattern was obtained after exposure to LNG and MIX. Regarding the phase I biotransformation genes, LNG and MIX increased *CYP3A27*’s expression but did not influence *CYP1A*’s expression. In humans, there is evidence of the involvement of CYP3A enzymes in the metabolisation of EE2 and LNG [8,83]. Regarding fish, there is a lack of either in vivo or in vitro studies exploring the effects of LNG on CYP3A27’s (and even on other CYPs’) expression, so more investigation is needed to understand effectively which of CYP3A’s isoforms are involved in LNG’s metabolisation in fish. The RTL-W1 cell line expresses *CYP1A*, but specifically for this cell line, 7-Ethoxyresorufin-*O*-Deethylase (EROD) induction (a measure of CYP1A activity) and CYP1A mRNA and protein levels have been mainly measured after exposure to polycyclic aromatic hydrocarbons (e.g., [84,85,86]). In vivo, CYP1A expression after LNG exposure was assessed only in a few studies [19,87,88]. In line with our result, Cardoso et al. [19] did not find differences between the liver protein expression of CYP1A of control and LNG-exposed zebrafish at either tested temperature (27 °C and 30 °C). However, in the three-spined stickleback kidney, *CYP1A* mRNA levels decreased after in vivo exposure to LNG [87,88], warning of differential impacts on the same gene across organs.

For phase II biotransformation genes, LNG increased the expression of *GST* in RTL-W1 at 21 °C (with an interaction effect with temperature) but did not impact the expression of *UGT*. *GST*’s expression was also upregulated in the bivalve zebra mussel (*Dreissena polymorpha*) digestive gland after 24 h of exposure to LNG (0.312, 3.12, and 6.24 μg/L) [89]. A decrease in liver GST enzyme activity was obtained in zebrafish after 7 days of exposure to 1 and 100 ng/L of P4 [37], reinforcing the ability of progestogens to interfere with the expression or activity of GST. In relation to UGT, Thibaut et al. [49] suggested that a phenol-type UGT and a testosterone (T)-type UGT exist in the RTL-W1 cell line, both responsible for the glucuronidation of T. Accordingly, *UGT* isoforms and gene families have been found in different fish species [90,91]. However, it is unknown if the identified isoforms metabolise EE2 or LNG, and we could not determine which isoform we amplified in the qPCR reaction. In human HEPG2 cells co-transfected with promoter regions of the UGT isoforms 1A1 and 2B7 and pregnane X receptor (PXR), P4 induced promoter activity and increased the mRNA levels of *UGT1A1* [92]. However, no effect was observed in *UGT2B7*, suggesting that P4’s action on *UGT1A1* was PXR-mediated and that different UGT isoforms may have substrate preferences. E2 could not induce the promoter activity of both *UGT1A1* and *UGT2B7*. Investigating the expression of PXR and various UGT isoforms in RTL-W1 cells in future studies and ascertaining their response to progestogens would be valuable.

*GST* was the only gene whose expression was influenced here by temperature, as it interacted with LNG exposure. *GST* mRNA levels were increased at 21 °C, compared with 18 °C. On the contrary, GSTs were downregulated in Atlantic salmon exposed for 43 days (in vivo chronic exposure) to 21 °C in relation to the control at 15 °C [30]. As Nuez-Ortín et al. [30] pointed out, GST downregulation is a common outcome of chronic heat shock, while, in line with our data, acute heat shock is usually associated with the upregulation of target genes. Regarding the other phase II enzyme, *UGT*’s expression was on the verge of increasing at 21 °C (*p* = 0.057). In female eelpout (*Zoarces viviparus*), hepatic microsomal UGT activity also showed some correlation with seasonal water temperature variations [93]. Our results and the literature support that phase II biotransformation processes can be the most affected by temperature. Moreover, in our study, this stressor showed the potential to modify how cells respond to LNG.

LNG and MIX upregulated the expression of *MRP2*. As an ATP-dependent efflux pump, MRP2 is responsible for excreting xenobiotics previously transformed into more polar compounds by, for example, GST and UGT. Some of the compounds transported through MRP2 include glutathione and sulphate drug/xenobiotic conjugates [94]. Particularly for estrogenic EDCs and LNG, conjugation with glutathione, catalysed by GST, is considered the most common detoxification mechanism [95]. Therefore, in this study, it is coherent that both *MRP2* and *GST* expression increased after LNG exposure, indicating an effort by RTL-W1 cells to detoxify and excrete LNG.

***CAT*** expression was also increased by LNG, aligning with in vivo studies in zebrafish where the CAT protein was increased by LNG (10 ng/L) at 27 °C and 30 °C [19]. *CAT* mRNA was also upregulated in the zebra mussel digestive gland after 7 days of exposure to LNG (0.312 and 6.24 μg/L) [89]. These increases in CAT after LNG exposure may signify that LNG leads to the formation of ROS [89]. Increases in CAT activity after 7 and 28 days of exposure to P4 (100 ng/L) have also been described in zebrafish liver [37], suggesting that this can be a common detoxification mechanism activated after exposure to progestogens.

Concerning *FAS*, only the MIX led to an increase in the mRNA expression of this particular target gene involved in lipid metabolism. This is a remarkable result, given the non-responses to EE2 alone. A previous study showed that estrogenic EDCs, in the same range of concentrations (µM), disrupted RTL-W1’s lipid metabolism and altered the mRNA expression of *FAS* and other lipid-related targets, at 24 and 48 h of exposure [53]. In our study, *FAS*’s expression only changed after exposure to the mixture of EE2 and LNG, suggesting an interaction between both compounds that should be explored in future studies expanding the gene portfolio. It would be particularly interesting to explore the pathways (genomic vs. non-genomic) involved in these effects. Interactions between EE2 and LNG were exposed earlier, e.g., with additive estrogenic effects reported for zebrafish brain aromatase gene expression, in vivo and in vitro [38]. The additive effects of EE2 and LNG (or other progestins) could be further explored in aromatase and other gene targets in RTL-W1 cells. Regarding *FABP1* and *FATP1*, in our in vitro model, their expression was not influenced by the compounds or temperature. In vitro, in 2D (1 µM) and three-dimensional (3D) (1–100 ng/L) cultured brown trout primary hepatocytes, EE2 exposure did not alter *FABP1*’s expression [66,96]. Nonetheless, in vivo, juvenile brown trout exposed to EE2 (50 µg/L) had significantly lower mRNA levels of *FABP1* [97]. Even aligning with other in vitro studies, our result here can be explained by the lack of a response of this in vitro model to EE2. However, it cannot be excluded, in our and other models, that other concentrations or exposure times could have produced different results. It is known that 3D models (including cell line-derived ones) maintain the original metabolic activity for longer periods [60,98]. It would be worth exploring if RTL-W1 cells in 3D were more responsive to EE2 and if *FABP1*’s expression can be modulated by EE2 (or other estrogens).

*FATP1*’s expression was previously induced in RTL-W1 cells after 24 h of exposure to BPA, which usually has an estrogenic mode of action [53]. As reported here, the basal *FATP1* levels were quite low [53]. It can be hypothesised that the BPA effect on *FATP1* mRNA levels could have happened through a signalling pathway other than the ER.

This work showed that the cell line expresses *HSP70b*, but this isoform did not respond to EDCs or temperature. HSP70 is implicated in the response to thermal and chemical stress [99,100]. The fact that *HSP70b* expression was stable across conditions may signify that neither tested factor caused enough protein stress to spark changes. Despite being environmentally realistic, our heat shock (+3 °C in relation to the growing temperature of the cells) is small compared to others reported in the literature that led to an increase in the expression of *HSP70b* [61,101]. Backing up this hypothesis, in the AOXlar7y cell line, derived from larvae of Atlantic sturgeon (*Acipenser oxyrinchus*), there were no alterations in *HSP70* gene expression kept at 25 °C, compared with the 20 °C control, but the 28 °C group differed from the other two groups [102]. So, a wider range of temperatures could be further tested to evaluate if *HSP70b* is responsive to temperature in the RTL-W1 cell line. Furthermore, exploring the gene expression of other HSPs from the diverse HSPs’ family in this cell line is a requirement to better disclose how temperature, EDCs, and other xenobiotics affect them.

From the main findings of this work, coupled with related but sparse literature data, it can be inferred that the RTL-W1 cell line, at least when cultured in a monolayer, is not well suited for studying estrogenic effects in fish liver. Nevertheless, it was remarkable that EE2 concurred with LNG to promote the upregulation of *FAS*, supporting using RTL-W1 to study unexplored mechanisms of the potential modulation of estrogens in lipid pathways. LNG impacted the gene expression of phase I, phase II, and other detoxification-related genes. These results suggest potential interferences of progestogens in fish liver biotransformation that are unexplored in the literature, calling for future studies to mechanistically explore the results obtained here. As the main regulator of detoxification genes, it seems relevant to prospectively analyse the expression of the aryl hydrocarbon receptor (AhR) in this cell line, as well as of other nuclear receptors implied in the liver response to xenobiotics, e.g., pregnane X receptor (PXR) or constitutive androstane receptor (CAR). Also, despite the relatively low range of temperatures tested, the cell line revealed changes in the gene expression of *GST* and even interaction effects of temperature and LNG. This interactivity is another topic worth exploring in this in vitro model to better characterise its usefulness.

## 5. Conclusions

The exposure to EE2 and LNG for 72 h, at 18 °C and 21 °C, had no impact on cell density and viability, nor did temperature alone. EE2 alone did not affect gene expression, confirming the cell line’s low estrogenic reactivity, at least in monolayer. In this vein, the cell line may be suitable or even ideal for exploring the mechanisms of action of compounds in an almost “estrogen-free signalling” cell environment. In contrast, LNG changed the expression of detoxification-related genes (*CYP3A27*, *MRP2*, *GST*, and *CAT*). Notably, the expression of the lipid metabolism gene *FAS* changed only in the MIX, denoting a synergic effect between EE2 and LNG. The PCA results corroborated the view, as the MIX primarily clustered with the LNG, secondarily with the EE2, and evidently diverged from C. The finding opens the possibility that despite the cells’ insensitivity to EE2, this chemical may have an impact when in mixtures. This prompts exciting questions and calls for further work about how those interferences happen. *GST* was the only gene whose expression differed among temperatures and was subjected to interactive effects between LNG and temperature. This study showed that RTL-W1 cells in a monolayer can be a system for studying isolated or combined effects of at least certain EDCs and temperature since some detoxification genes were susceptible to the impacts of LNG. Because this study was limited to 72 h of post-exposure, future work could cover a range of exposure times, as changes in expression could exist at different time points. The study contributed to better qualifying the RTL-W1 cells’ reactivity to estrogens, progestogens, and temperature.

## Figures and Tables

**Table 1:** Primer sequences of the target and reference genes with the detailed protocol, including the annealing temperatures and amplification efficiencies (E) in bold.

Gene *	Primer (5′-3′)	Protocol	E (%)	Reference
*CYP1A*	F: GATGTCAGTGGCAGCTTTGA R: TCCTGGTCATCATGGCTGTA	95 °C—3 min (95 °C—20 s; **60.0 °C**—20 s; 72 °C—20 s) 40× 95 °C—1 min	**97.7**	[60]
*CYP3A27*	F: GACGGTGGAGATCAACG R: GAGGATCTCGACCATGG	95 °C—3 min (95 °C—20 s; **60.0 °C**—20 s; 72 °C—20 s) 40× 95 °C—1 min	**99.0**	[60]
*HSP70b*	F: AGGCCCAACCATTGAAGAGA R: GCAATGTCCAGCAATGCAATA	95 °C—3 min (95 °C—10 s; **60.0 °C**—30 s; 72 °C—30 s) 40× 95 °C—1 min	**92.2**	[61]
*UGT*	F: ATAAGGACCGTCCCATCGAG R: ATCCAGTTGAGGTCGTGAGC	94 °C—3 min (94 °C—20 s; **60.0 °C**—20 s; 72 °C—20 s) 40× 94 °C—1 min	**94.9**	[60]
*GST*	F: AGCTGCTCCCAGCTGATCC R: CAAACCACGGCCACATCATGTAATC	94 °C—3 min (94 °C—20 s; **60.0 °C**—20 s; 72 °C—20 s) 40× 94 °C—1 min	**93.9**	[62]
*CAT*	F: CACTGATGAGGGCAACTGGG R: CTTGAAGTGGAACTTGCAG	95 °C—3 min (95 °C—10 s; **58.0 °C**—30 s; 72 °C—30 s) 40× 95 °C—30 s	**104.5**	[63]
*MRP2*	F: CCATTCTGTTCGCTGTCTCA R: CTCGTAGCAGGGTCTGGAAG	94 °C—3 min (94 °C—20 s; **60.0 °C**—20 s; 72 °C—20 s) 40× 94 °C—1 min	**100.2**	[60]
*ERα*	F: GACATGCTCCTGGCCACTGT R: TGGCTTTGAGGCACACAAAC	95 °C—4 min (95 °C—1 min; **61.6 °C**—1 min) 40× 95 °C—1 min	**95.6**	[64]
*VtgA*	F: AACGGTGCTGAATGTCCATAG R: ATTGAGATCCTTGCTCTTGGTC	95 °C—3 min (95 °C—10 s; **62.9 °C**—1 min) 40× 95 °C—30 s	**102.5**	[64]
*FAS*	F: ACCGCCAAGCTCAGTGTGC R: CAGGCCCCAAAGGAGTAGC	95 °C—3 min (95 °C—20 s; **60.0 °C**—20 s; 72 °C—20 s) 40× 95 °C—1 min	**95.0**	[65]
*FABP1*	F: GTCCGTCACCAACTCCTTC R: GCGTCTCAACCATCTCTCC	94 °C—3 min (94 °C—20 s; **57.0 °C**—20 s; 72 °C—20 s) 40× 95 °C—1 min	**102.5**	[66]
*FATP1*	F: AGGAGAGAACGTCTCCACCA R: CGCATCACAGTCAAATGTCC	95 °C—3 min (95 °C—20 s; **60.0 °C**—20 s; 72 °C—20 s) 40× 95 °C—1 min	**92.9**	[67]
*β-act*	F: TCTGGCATCACACCTTCTAC R: TTCTCCCTGTTGGCTTTGG	94 °C—3 min (94 °C—20 s; **55.0 °C**—20 s; 72 °C—20 s) 40× 94 °C—1 min	**99.7**	[66]
*ef1α*	F: TGCCACACTGCTCACATC R: TCTCCAGACTTCAGGAACTTG	94 °C—3 min (94 °C—20 s; **55.0 °C**—20 s; 72 °C—20 s) 40× 94 °C—1 min	**97.4**	[68]

* *CYP1A* (*Cytochrome P450 1A*), *CYP3A27* (*Cytochrome P450 3A27*), *HSP70b* (*Heat Shock Protein 70 b*), *UGT* (*Uridine diphosphate* (*UDP*)*-glucuronosyltransferase*), *GST* (*Glutathione S-transferase omega 1*), *CAT* (*Catalase*), *MRP2* (*Multidrug Resistance-Associated Protein 2*), *ERα* (*Estrogen Receptor α*), *VtgA* (*Vitellogenin A*), *FAS* (*Fatty Acid Synthase*), *FABP1* (*Fatty Acid Binding Protein 1*), *FATP1* (*Fatty Acid Transport Protein 1*), *β-act* (*β-actin*), *ef1α* (*Elongation Factor 1 α*).

## Data Availability

Within reasonable use limits, the data can be made available from the corresponding authors.
